# 1897. Therapeutic Drug Monitoring in CSF and Plasma in Tuberculous Meningitis:Correlation with Disease Outcome

**DOI:** 10.1093/ofid/ofad500.1725

**Published:** 2023-11-27

**Authors:** Urvashi Singh, Adarsh Aayilliath

**Affiliations:** All India Institute of Medical Sciences ,New Delhi, New Delhi, Delhi, India; All India Institute of Medical Sciences ,New Delhi, New Delhi, Delhi, India

## Abstract

**Background:**

Tuberculous meningitis (TBM) is a serious form of extra-pulmonary tuberculosis with significant morbidity and mortality. TBM patients are treated with pulmonary tuberculosis regimen, often requiring extension for 1 year or longer. Current study was designed to find any association of CSF and blood levels of anti-tubercular drugs with treatment response in patients with tuberculous meningitis using therapeutic drug monitoring.

Pharmacokinetic Parameters of Rifampicin and Isoniazid in CSF and Plasma

**Figure:**
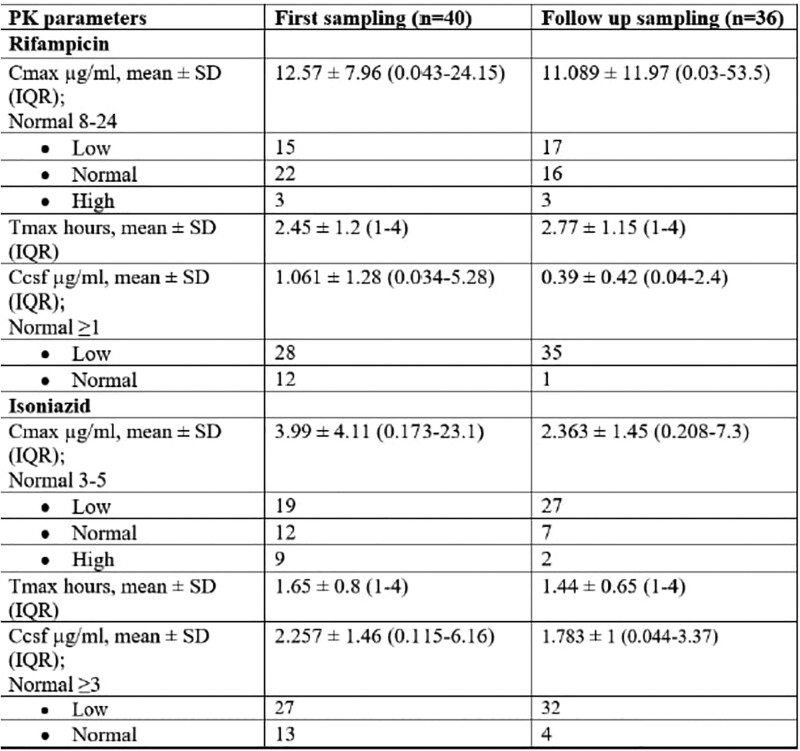

**Methods:**

A prospective observational study was conducted in a tertiary care institute in North India among clinic-radiologically and/or microbiologically diagnosed TBM patients. Plasma and CSF levels of anti TB drugs (rifampicin, isoniazid, pyrazinamide and ethambutol) were determined using Liquid Chromatography Mass Spectrometry technology at 1 week and 1 month (follow up) after initiating treatment. The primary outcome assessed was association of drug levels with treatment response. The secondary outcomes assessed were association of drug levels with treatment duration/alteration, patient demographic profile, comorbidities and adverse effects. Patients were followed-up till treatment completion.

**Results:**

A total of 40 patients with TBM were recruited. All received daily weight-based doses of anti TB drugs. Low Cmax of INH and rifampicin in plasma were observed in 60.5% & 42.1% of observations respectively. In CSF, low Cmax of INH and rifampicin were observed in 77.6% & 82.9% of observations respectively. The concentrations of INH (4 hours after drug intake) during follow up were significantly lower among patients with poor response, at 1 month (p=0.008) & 6 months (p=0.009). Similarly, low Cmax & CSF levels of INH at follow up sampling were significantly associated with poor response at 6 months of therapy (p= 0.022 & 0.042 respectively).

**Conclusion:**

Low plasma and CSF concentrations of isoniazid were associated with a poor response to treatment in patients with TBM. Therapeutic drug monitoring can guide dose modification of ATT to achieve target concentrations in patients with TBM and facilitate optimal treatment outcomes. Higher dose INH can be considered in TBM treatment regimen for better outcome.

**Disclosures:**

**All Authors**: No reported disclosures

